# Buttermilk

**Published:** 1898-02

**Authors:** 


Buttermilk which at one time was thought only fit for the hogs, as its virtues are better known is eagerly sought after as not only a healthy but a very pleasant drink, especially by the dyspeptic and old people. Down in the vicinity of Wall street the other day we noticed a stand on the corner of Liberty street, around which several old men, most of them millionaires, were gathered, drinking great glasses of rich, iced buttermilk. This, one of them said, was his lunch, and he often came down town to get his drink. The lactic acid dissolves the phosphate of lime and keeps the blood in good condition, thereby preventing or retarding that ossification of tendons and arteries so common in old people.—*N. Y. Med. Times.*

